# Professional Unity—Medical Societies

**Published:** 1871-02

**Authors:** 


### PART IV.

### Editorials, Reviews, Items and News.

### PROFESSIONAL UNITY—MEDICAL SOCIETIES.

Order is the first and great law of the Universe. When we look around, above and beneath us, we find a combination of elements and forces, harmoniously co-operating for the accomplishment of one grand design, glorious in conception and perfect in details. The revolution of the suculent juices of the tiny plant, completing its round from rootlet to flower; the mighty cycles measured by the siderial Heavens,—of planets and suns—proclaim the law of harmony and order. Nature's great author, by laws immutable and everlasting, has impressed perfection upon all His works; and these laws—revealing the mind of the sovereign Architect—far more than the things created, declare the wisdom, power and love, of Nature's God.

As a law—pervading all things, both material and spiritual,—we find association and union of forces, universal—from the minute atoms floating in our atmosphere, to the myriad hosts of angelic beings surrounding the throne of the everlasting Father.

Man—immortal in spirit; ennobled by powers well nigh angelic, and made in the image of his Creator—has ever found the only source of true happiness, to spring from a ready, willing, cheerful obedience to law. In all the dominions of nature, both material and spiritual, law must assert its supremacy. Obedience to law brings its rewards: the violation of law entails ruin and disaster, and its penalties, at last, become a law of retribution.

Human wisdom too often exhausts its power, blights its purpose, and insults its Creator, in its feeble efforts to override His will, or subvert His decrees. History photographs its follies and paints its delusions. Its pages groan with examples of nations overthrown—of their utter destruction and annihilation—as the legitimate but terrible results, of the violation of known laws, streaming in living light from earth and sky, or engraven upon the hearts and consciences of men.

As Medical Journalists, in penning the above, we do not desire to overstep the bounds of our legitimate sphere. Science is, truly, the handmaid of religion; and if we could succeed in awakening in the hearts of our professional brethren a sense of their deep obligations to religion, as the foundation and glory of Science, we should not occupy their time in vain. If an irreligious astronomer be declared mad, what shall be said of an irreligious physician? The one gazes, untouched, upon the sublime panorama of the skies—of revolving orbs and resplendent suns. The other looks unmoved upon the *chef d'œuvre* of nature, eternal in essence, and an heir elect of the Creator's power and glory!

Purity of life, probity of heart, and a willing, cheerful—yea, inflexible—adherence to truth, based upon the grand moral platform of justice and right, as drawn from revealed religion, are duties common to mankind. The physician, conscious of his obligations, true to his manhood, and just to his science, cannot neglect or evade them, without marring his nature and dishonoring his cause.

The law of association and union of forces, pervades society no less than nature. To attempt its proof would be superfluous, and an insult to the intelligence of our readers. To know a fact, and then to utilize that fact, are, however, two very different propositions. Usually, mankind know far more than they are willing to perform. Indeed, we had better be ignorant of a duty, than after knowing it, fail to recognize or perform it.

Prompted by feelings which we ought not suppress, we have written the above as an introduction, to show the importance and necessity of professional unity. The union of medical forces—of its members—is no less imperative than those of States and Nations, if those interested expect to give power and influence to the profession. It is a law, the violation of which, will inevitably bring the penalties of dishonor and ruin upon all. The profession throughout the country is devitalized, and has lost its influence upon the minds and hearts of the people. There was a time when the hearts of the populace were swayed by the power of the profession—when, to be a Physician, was an appellation of honor and distinction. How is it now? Need we tell our readers it is as barren of power as the title is of honor? They have only to look around them and bear witness to its truth. The true interests of the profession have been paralyzed by discord and rivalry; by forced, hot-bed medical educations; by isolation and selfish greed for gain, and by departures from, and violations of, the only true ethics of the profession.

There is a remedy for all this. Shall it be applied? It only requires unity of purpose, and obedience to law to restore it to its pristine power and glory. How ought it to be done?

1st. Bring the elements of the profession in close, harmonious contact. Let the great law of association and union of forces have its perfect work in every department of our science. To do this individuals must respond to its demands.

Have any violated the law? Let them return to it. Have any allowed selfish greed to seduce them from the right? Let them renounce their errors. Let there be an upheaval in the professional valley of dry bones. Soon, bone shall come to bone, muscle to muscle; and the beautiful fabric of Science resurrected, will lift itself erect, the pride and glory of all.

2d. Let Physicians everywhere unite—upon the basis of justice and truth. Cast away private enmities and jealousies. Encourage the weak by appealing to the heart; by showing that principle is the only pathway to honor and success. Hold up the hands of the strong—as the pillars of the truth. Rally around the ensign of the profession—the "Standard of Right"—the Ethics. Let the law mete out its penalties, without partiality or prejudice, to all violators..

3d. Organize! Organization is the bow of promise to the profession. It is nature's law—it is the christian's law—it is the law of Heaven! Then, let the friends of Science, everywhere, organize Medical Societies. Found them upon the truth. Great, good and wise men have furnished a model. Let that model be the "corner stone"—the foundation. Build upon it.

Having done this—having obeyed the great law of association and union of forces—then let the light of medical truths flood the land. Let the love of Science kindle a glow in every heart. Gather facts—and report them to the medical journals for the good of all. Cast them upon the waters, and they will return to enrich the giver. Then the profession will be honored in honoring itself; then quackery will hide its face abashed, and our noble art, clothed in the habiliaments of peace, truth and mercy, will enthrone itself the Queen of Science, loved by her subjects and honored by the world.

